# Body image distress in head and neck cancer patients: what are we looking at?

**DOI:** 10.1007/s00520-020-05725-1

**Published:** 2020-09-03

**Authors:** H. C. Melissant, F. Jansen, S. E. Eerenstein, P. Cuijpers, E. Laan, B. I. Lissenberg-Witte, A. S. Schuit, K. A. Sherman, C. R. Leemans, I. M. Verdonck-de Leeuw

**Affiliations:** 1 Amsterdam UMC, Vrije Universiteit Amsterdam, Department of Otolaryngology–Head and Neck Surgery, Amsterdam Public Health Research Institute, Cancer Center Amsterdam, P.O. Box 7057, 1007 Amsterdam, MB Netherlands; 2 Cancer Center Amsterdam (CCA), Amsterdam UMC, Vrije Universiteit Amsterdam, Amsterdam, The Netherlands; 3 Department of Clinical, Neuro- and Developmental Psychology, Faculty of Behavioral and Movement Sciences, Amsterdam Public Health Research Institute, Vrije Universiteit Amsterdam, Amsterdam, The Netherlands; 4 Department of Sexology and Psychosomatic OBGYN, Amsterdam UMC, Academic Medical Center, Amsterdam, The Netherlands; 5 Department of Epidemiology and Biostatistics, Amsterdam UMC, Vrije Universiteit Amsterdam, Amsterdam, The Netherlands; 6 Centre for Emotional Health, Department of Psychology, Macquarie University, Sydney, Australia

**Keywords:** Body image, Health-related quality of life, Head and neck cancer, Depression, Psychological distress, Prevalence

## Abstract

**Purpose:**

The aim of the present study is to investigate the prevalence of body image distress among head and neck cancer (HNC) patients after treatment and to examine its association with sociodemographic and clinical factors, health-related quality of life (HRQOL), HNC symptoms, sexuality, self-compassion, and psychological distress. Second, we aim to explore daily life experiences of HNC patients regarding body image.

**Methods:**

A cross-sectional survey among HNC patients investigated the prevalence of body image distress based on the Body Image Scale. Multivariable logistic regression analysis was applied to study associations with sociodemographic and clinical factors, HRQOL (EORTC QLQ-C30), HNC symptoms (QLQ-HN43), sexuality (FSFI-6; IIEF-5), self-compassion (SCS-SF), and psychological distress (HADS). Qualitative data from a body image writing intervention was used to explore experiences in daily life related to body image.

**Results:**

Body image distress was prevalent in 13–20% (depending on cut-off scores) of 233 HNC patients. Symptoms of depression (*p* < 0.001), younger age (*p* < 0.001), problems with social contact (*p* = 0.001), problems with wound healing (*p* = 0.013), and larger extent of surgery (*p* = 0.014) were associated with having body image distress. This model explained 67% of variance. Writing interventions of 40 HNC patients showed that negative body image experiences were related to appearance and function, with social functioning problems described most often.

**Conclusion:**

Prevalence of body image distress in HNC patients, using different cut-off scores, is 13–20%. Younger patients, patients after extensive surgery, and patients who had wound healing problems are most at risk. There is a significant association between body image distress and depressive symptoms and social functioning.

**Electronic supplementary material:**

The online version of this article (10.1007/s00520-020-05725-1) contains supplementary material, which is available to authorized users.

## Introduction

Head and neck cancer (HNC) patients have to deal with a wide range of symptoms related to HNC cancer and its treatment [1]. Vital functions can be affected, such as breathing, speaking, and swallowing. These functional impairments may negatively influence a patient’s body image [2]. Also, appearance changes in the visible head and neck area may influence body image [3]. Surgical treatment may cause scarring, an amputated facial area, an affected facial contour and expression, or result in a tracheostomy [4–6]. Radiotherapy may induce swelling, fibrosis, and alterations in skin pigmentation [5].

Body image is defined by thoughts, feelings, and perceptions about the body and its functions [7]. A previous review identified nine studies that reported the prevalence of body image distress among HNC patients [5], with prevalence rates ranging from 25 to 77%. The lowest prevalence was found among patients after treatment of oral or oropharyngeal cancer [8] and the highest among newly diagnosed oral cancer patients [9]. Studies mainly focused on a specific HNC subsite (oral/oropharyngeal cancer) or a specific treatment modality (surgery). Information is scarce on body image distress in patients with other HNC sites, and patients treated with (combinations of) surgery, radiotherapy, and chemotherapy.

Furthermore, more data are needed to understand which factors are associated with body image distress and how it affects daily life in HNC patients. Body image distress is found to be associated with decreased health-related quality of life (HRQOL) and symptoms of depression in HNC patients [10–12]. In addition, it may affect their identity and social relationships [6]. Body image distress may also be related to sexual problems, for example, because HNC patients no longer feel sexually attractive [4].

Previous qualitative research has described how patients with amputations in the face (e.g., nose or eye) experience and adjust to a changed appearance after HNC. In daily life, patients are constantly reminded of their disfigurement, evoked by painful or itching sensations or by unwanted attention from others [13]. Patients seem to gradually learn to cope with these situations [13, 14]. However, insight into experiences from HNC patients with other (more common) bodily changes than an amputation is warranted.

The first aim of the present study is to investigate the prevalence of body image distress in HNC patients, and whether sociodemographic and clinical factors, HRQOL, HNC symptoms, sexuality, self-compassion, and psychological distress are associated with body image distress. The second aim is to qualitatively analyze experiences of HNC patients that caused negative feelings about themselves and their body, and to explore thoughts and feelings that accompany these experiences. Results of the present study will provide more insight in what body image distress means to HNC patients, and this will facilitate supportive care targeting HNC patients with body image distress.

## Methods

### Study design and participants

This mixed-methods study entailed a quantitative cross-sectional survey among HNC patients and qualitative analyses of writing using a writing prompt among patients with an identified need for body image care.

HNC patients were invited to participate in a written survey on the prevalence of body image distress. Patients were recruited at the Department of Otolaryngology–Head and Neck Surgery of Amsterdam UMC, location VUmc. HNC patients were eligible if they (1) received treatment for HNC (all tumor sites, all treatment modalities) with curative intent, (2) completed treatment 6 weeks to 5 years prior, and (3) provided written informed consent. HNC patients were excluded if they were < 18 years, had cognitive impairments, were unable to read and write Dutch, or participated in a prospective cohort study [15]. From September 2018 to September 2019, eligible HNC patients received an invitation for this study from their physician.

For the qualitative part of the study, HNC patients who completed the survey could participate in a separate consecutive study investigating a writing intervention that aims to reduce body image distress. Patients were asked to participate if they suffered from negative thoughts, feelings, and experiences regarding their changed body, and had a need for care to improve their body image. HNC patients who participated signed a separate informed consent form and subsequently received the intervention (booklet or web-based version). After finishing the writing intervention, patients were asked to return (a copy of) their writings to the researcher. The intervention “My Changed Body” is a self-paced writing activity [16]. We used respondents’ answers on the first writing prompt, in which they were asked to describe a negative event that they have experienced about their body after having undergone an HNC treatment, for example, an event that involved failure, humiliation, or rejection. Respondents are asked to describe the event and provide details regarding what led up to it, who was present, what happened, and how he/she felt and behaved at the time. The study was approved by and conducted according to regular procedures of the local ethical committee of VU University Medical Center. All participating patients provided informed consent.

### Outcome measures

Clinical characteristics were retrieved from medical files. The survey included items on sociodemographic characteristics and patient-reported outcome measures (PROMs).

The primary outcome was the 10-item Body Image Scale (BIS), measuring affective, behavioral, and cognitive body image symptoms. It was developed for use in oncology populations [17]. Items are answered on a scale ranging from 0 “not at all” to 3 “very much.” A total score (range 0–30) can be calculated by summing up the items, with higher scores indicating a higher level of body image distress. The BIS has shown adequate psychometric properties [18] and is translated and validated in Dutch [19].

HRQOL was measured with the EORTC QLQ-C30, a cancer-specific quality of life questionnaire [20], and HNC symptoms were measured using the EORTC QLQ-HN43, a module specifically designed for HNC patients [21]. Sexuality was measured with the 6-item Female Sexual Function Index (FSFI-6) [22] for women and 5-item International Index of Erectile Function (IIEF-5) [23] for men. Patients were categorized in the “no sexual activity” group if they reported not to have had sexual activity and intercourse in the past 4 weeks. Validated cut-off scores [22, 23] for women (cut-off 19) and men (cut-off 21) were used to classify patients either as having reported sexual problems or not, to enable cross-gender analyses. To measure self-compassion, the 12-item Self-Compassion Scale-Short Form (SCS-SF) was used [24]. Lastly, psychological distress was measured using the total score of the 14-item Hospital Anxiety and Depression Scale (HADS), and two subscales that measure anxiety (HADS-A) and depression (HADS-D) [25]. All instruments used in the present study are validated [21, 24, 26–29].

### Statistical analyses

Descriptive statistics were generated for sociodemographic and clinical characteristics and the prevalence rate. The prevalence of body image distress was calculated using the most often used BIS cut-off points ≥ 10 [17] and ≥ 8 [30]. To investigate potential factors associated with body image distress (BIS cut-off point ≥ 8), logistic regression analyses were used. A multiple logistic regression model with a stepwise forward selection procedure was applied to investigate which factors were significantly associated with body image distress. Based on univariate logistic regression analyses, variables with *p* value for entry < 0.05 were added sequentially to the multiple regression model. Potential sociodemographic factors included age, gender, relationship status, education level, and work situation. Clinical factors included tumor site, tumor stage, HPV status, time since treatment, treatment modality, surgical reconstruction, neck surgery, and extent of surgery (see Supplementary file 1 for variable categories). Included PROMs were the QLQ-C30 summary score [31], QLQ-HN43 subscales and single items, sexuality (no activity, sexually active without and with sexual problems), the SCS-SF total mean score, and the HADS total score and subscales.

To demonstrate a body image distress prevalence of 25% (based on need for support regarding body image distress [32]), and with a 95% CI of a prevalence between 17.5 and 32.5%, 139 patients were needed for the present study. For all analyses, a standard alpha level of 0.05 was used. Analyses were carried out using SPSS version 26 (IBM Corp., Armonk, NY).

### Qualitative analysis

Thematic analyses were undertaken by two researchers trained in qualitative analysis (H.M. and A.S.) [33]. The coders first familiarized themselves with the data, then initial codes were identified, and underlying themes were explored. After reviewing initial findings, data were categorized into key issues and themes. Data were analyzed individually and after each phase, findings were discussed in consensus meetings. Supplementary file 2 presents the COREQ criteria checklist for describing and reporting the qualitative analysis procedures and findings [34].

## Results

### Study sample

In total, 521 HNC patients were invited to participate in the study of which 233 patients (45%) participated. Of these patients, 76 participated in the writing intervention study, of whom 40 returned their writing, and 29 had relevant quotes about their changed body. Patient characteristics are presented in Table 1.

**Table 1:** Patient characteristics

Characteristics	*N* (%)	
	Total sample (*n* = 233)	Qualitative sample (*n* = 40)^a^
Mean age in years (SD)	67 (10.7)	66 (10.1)
Gender		
Male	154 (66)	28 (70)
Female	79 (34)	12 (30)
Married/in a relationship		
Yes	172 (74)	30 (75)
No	61 (26)	10 (25)
Education level		
Lower	47 (20)	11 (28)
Middle	111 (48)	19 (48)
Higher	75 (32)	10 (25)
Work situation		
Employed	68 (29)	11 (28)
Unemployed/retired	165 (71)	29 (73)
Tumor site		
Oral cavity	51 (22)	9 (23)
Oropharynx	57 (25)	9 (23)
Hypopharynx	12 (5)	1 (3)
Larynx	64 (28)	13 (33)
Other^b^	49 (21)	8 (20)
Tumor stage		
Stage I/II	103 (44)	14 (35)
Stage III/IV	120 (52)	23 (58)
Unknown	10 (4)	3 (8)
HPV positive (in case of oropharyngeal cancer)	40 (70)	7 (78)
Time since treatment in years (median) (IQR)	3.3 (2.2–4.5)	3.5 (2.5–4.8)
Single treatment	111 (48)	16 (40)
Surgery	62 (56)	7 (18)
Among which CO_2_ laser	33 (53)	5 (71)
Radiotherapy	49 (44)	9 (23)
Combination treatment	122 (52)	24 (60)
Chemoradiotherapy	51 (42)	9 (23)
Surgery and (chemo)radiotherapy	70 (57)	15 (38)
Other^c^	1 (0.8)	0 (0)
Reconstruction^d^		
None	45 (34)	6 (27)
Primary closure	47 (35)	10 (46)
Surgery with reconstruction	41 (31)	6 (27)
Neck surgery^d^		
Yes	62 (47)	11 (50)
No	71 (53)	11 (50)
Surgery extent^e^		
Small	37 (28)	5 (23)
Moderate	30 (23)	5 (23)
Large	36 (27)	7 (32)
Very large	30 (23)	5 (23)

*IQR* interquartile range

^a^*n* = 29 had relevant quotes about their changed body

^b^Parotid *n* = 22, skin tumor head–neck region *n* = 7, nose and paranasal sinus *n* = 8, nasopharynx *n* = 6, unknown primary *n* = 5, osteosarcoma *n* = 1

^c^Other combination treatment was surgery with chemotherapy

^d^Only those patients who had a surgical treatment

^e^Small: CO_2_ laser of vocal fold, lip excision, ear amputation, skin excision small nose tumor. Moderate: excision of sublingual/submandibular salivary gland, transoral excision, lip surgery with reconstruction, partial sinus resection, skin excision with local reconstruction, neck surgery. Large: parotidectomy with neck surgery, marginal and segmental mandibular resection, transoral excision with reconstruction, extensive sinus surgery, maxillectomy, skin excision with neck surgery or reconstruction. Very large: commando procedure, laryngectomy, lateral temporal bone surgery

### Prevalence of body image distress and associated factors

The prevalence of body image distress was 13% (cut-off ≥ 10) to 20% (cut-off ≥ 8) (median = 2, IQR = 0–6). Univariate logistic regression analyses showed that age, gender, education level, treatment modality, surgery extent, EORTC QLQ-C30 summary score, all QLQ-HN43 subscales, self-compassion, and psychological distress were significantly associated with body image distress (Supplementary file 1). The multiple logistic regression model showed that five factors were significantly and independently associated with body image distress: symptoms of depression, younger age, problems with social contact, problems with wound healing, and larger extent of surgery (Table 2). The model explained 67.0% (Nagelkerke *R*^2^) of the variance in body image distress.

**Table 2:** Results of the multivariate logistic regression analyses

Variable	OR (95% CI)	Significance
HADS depression	1.45 (1.19–1.77)	< 0.001
Age	0.87 (0.81–0.94)	< 0.001
Problems with social contact	2.82 (1.54–5.18)	0.001
Problems with wound healing	1.66 (1.11–2.48)	0.013
Surgery extent		0.014
Very large	1	
Large	0.08 (0.01–0.59)	
Moderate	0.02 (0.00–0.25)	
Small	0.22 (0.03–1.45)	

### Qualitative responses

The writing in the intervention showed that negative body image experiences were related to appearance changes and (dys)function (Table 3). Categories of (dys)function included psychological, daily, social, physical, and occupational functioning, and functioning in an intimate relationship [35].

**Table 3:** Negative experiences related to bodily changes after HNC

Topic	Key issues	Themes
Appearance changes	Visible changes	Looking tired and worn out
		Neck is dented and mouth is asymmetric
		Severe weight loss
		Body has grown old quicker
		(Ugly) scars
		Burned skin due to radiotherapy
	Non-visible changes	Changes are invisible from the outside
Psychological functioning	Identity threat	Feeling lonely and sad after rejection as blood donor
		Feeling sad after losing typical generous laughter
		Losing trust in own body
	Shame	(Temporarily) feeling ashamed for burned skin at throat
		Changed face because of scars and edema
		Not daring to face people because of changed appearance
	Sadness, depression	Feeling depressed about losing vocal cords
		Feeling awful because of physical disability (concerning the tongue)
	Feeling bad and ugly	Praying to die right after surgery
Daily functioning	Low energy level	It takes much time to be able to function normally again
		Fatigue/sleeping much
Social functioning	Eating (in public)	Embarrassing situation
		Social isolation due to problems with eating, drinking, and speech
		Difficulties with social activities due to problematic combination eating and talking
	Talking (in public)	Talking is bothersome because voice sounds nasal
		Getting frustrated if others cannot hear patient
		Speaking loudly in noisy environment is problematic because stoma plaster does not hold
		Slurring as a result of surgical procedure is uneasy because of alcoholic past
		Fear of talking in public after laryngectomy
		Hoarse voice is problematic
	Reaction from others	Being ignored because of unusual voice
		Others do not know how to react to uneasy situation
		Feeling stared at while doing grocery shopping
		Visitors think slime and drool from patient is filthy
		Others do not dare to ask how patient is doing
		Feeling misunderstood if others compare their fatigue with cancer-related fatigue
Physical functioning	Practicing a hobby	Physical recovery to be able to play golf again takes much effort
		Feelings of loss because patient cannot sing anymore
	Going on holiday	Considering to cut short holiday because of physical symptoms
Occupational functioning	Changes at work	Feeling rejected and superfluous
		Becoming unfit for work is heavy news
		Suspicion that cooperation is canceled due to changed appearance
Functioning in intimate relationships	Rejection	Being let down by partner
	Conflict	Revealing illness to others without patient’s consent
		Feeling like a burden to partner

#### Appearance changes

Some patients (*n* = 7) described visible changes in their appearance, for example, having a dented neck or an asymmetric mouth. One patient explained: “I look a bit older, around my chin some deep furrows have emerged and my lips aren’t so pronounced anymore.”

#### Psychological functioning

Several patients (*n* = 6) put emphasis on feelings of shame, depression, and feeling bad and ugly. Another issue mentioned was a threatened identity (*n* = 3). Something that belonged to their identity was taken away, like being rejected as a blood donor, or having a typical laugh: “In particular, I feel sad when I realize that I cannot sing anymore and that my generous laughter (the sound) is gone. I miss that enormously.”

#### Daily functioning

Some patients reported that bodily changes had a negative impact on their daily life (*n* = 6), in particular regarding their energy level: “In the beginning the energy level of my body bothered me. In my experience, it took a long time before I could function ‘normally’ again: sporting, working, living.”

#### Social functioning

Many patients (*n* = 12) wrote about the impact of their changed body on their social life. Difficulties with eating in public were frequently mentioned (*n* = 6). It could cause embarrassing situations: “Fluids and food come out of my nose if I don’t pay close attention. This can be very bothersome, especially in company. I always need to have a handkerchief ready when I eat something.”

A related topic was talking in public (*n* = 4). The different sound of voice (hoarse, nasal) or having a voice prosthesis caused difficulties with intelligibility, which was frustrating or shameful for some. “Ever since the surgery, I have the feeling that I am slurring. Given my alcoholic past, I don’t feel comfortable with that.”

Some patients (*n* = 5) were bothered by reactions of others to their changed body. Other people do not always know how to react to patients’ changed appearance or dysfunction. “I was in the grocery store and a boy around nine years old was staring at me. That’s nothing out of the ordinary, as it happens on a daily basis. But then, he drew his mother’s attention to me and she started to stare at me extensively, it was very bothersome.”

#### Physical functioning

For some patients (*n* = 3), physical dysfunction complicated participation in activities or hobbies, for example, not having the physical fitness to play golf. “It took around nine months before my physical condition was good enough to be able to golf 18 holes again. […] During that time, there are a lot of moments when you feel bad and sad.”

#### Occupational functioning

Some patients (*n* = 3) described how they became unfit for their occupation or had to deal with negative consequences: “An organization, which I already represented over 30 years, canceled the contract with me after a management change. It wasn’t said that it had to do with my appearance, but I saw one of the directors look at me very critically/disapprovingly.”

#### Functioning in intimate relationships

A few HNC patients (*n* = 2) wrote about relationship problems. For example, a patient was let down: “I was so sad when I was let down by my partner during my stay in the hospital. I really felt rejected.”

## Discussion

In the present study, the prevalence of body image distress among HNC patients was 13–20%. Body image distress was significantly associated with symptoms of depression, younger age, problems with social contact, problems with wound healing, and larger extent of surgery. Patients who participated in a writing intervention reported that negative body image experiences are related not only to changes in appearance but also in functioning, including psychological, daily, social, physical, occupational functioning, and functioning in an intimate relationship.

The prevalence rate in the present study was lower compared with previous studies in the head and neck cancer context, which range from 25 to 77% [5]. A wide variety of instruments (e.g., Derriford Appearance Scale, Body Image Survey, BIS) used to assess body image could explain this discrepancy. The highest prevalence in previous studies of 77% was found among newly diagnosed oral cancer patients who reported future appearance concerns in a clinical interview [9]. This may be more related to fear or expectations than existing body image problems. If only BIS outcomes are compared, comparable levels of body image distress were found [36, 37]. In a study among HNC patients, for instance, < 15% had a BIS score higher than 9 [36], and in a study among female HNC patients, the mean overall BIS score was 4.50 [37].

Results of the present study show that patient characteristics, social factors as well as psychological factors are associated with body image distress. This is consistent with a conceptual framework on causal factors, moderators, and sequelae of body image in HNC patients [5]. In addition, the explained variance of the model in the present study is higher than in a previous study where disease stage, gender, and depression explained 32% of the variance [9]. An explanation may be that our study included quality of life and clinical variables, suggesting that difficulties with wound healing, problems with social contact, and extent of surgery are key factors associated with body image distress.

Extent of the surgical procedure was related to body image distress in the present study, in contrast with a study from Chen et al. [38] who found that the surgical procedure did not influence body image. These conflicting results could be explained by the different study sample used. Inclusion of patients treated with CO_2_ laser (less extensive surgery) in the present study might explain lower body image distress compared with patients who had a commando procedure (a major operation involving removal of facial structures) or total laryngectomy. In the study sample of Chen et al. [38], the majority of patients received very extensive surgery: total/partial laryngectomy or oral excision with facial reconstruction.

The association between body image distress and depression in HNC patients was also found in studies among newly diagnosed HNC patients [9] and HNC patients from diagnosis until 12 weeks post-treatment [12]. Our study provides evidence that the association between body image distress and depression is also present for a longer time after treatment. Feelings of loss associated with a changed appearance may explain this association [12].

There was also a significant association between problems with social contact and body image distress. This outcome was further confirmed by the results of our qualitative analysis which showed that eating in public, talking in public, and reactions from others were frequently mentioned events that triggered body image distress. A previous qualitative study among HNC patients also describes social concerns and avoiding people because of body image distress [39]. Over time, HNC patients are at risk to become socially isolated if no active coping strategies are undertaken [40]. HNC patients who have speech and eating problems report highest levels of social avoidance [2].

In the univariate regression analysis, a statistically significant inverse association between body image distress and self-compassion was found in HNC patients. This is in line with previous research among breast cancer patients, which has shown that self-compassion is inversely related to body image distress [41]. Self-compassion may protect against a negative judgment of one’s post-cancer body, e.g., by being kind to oneself.

The qualitative analysis in the present study revealed that identity was an important aspect of body image. HNC patients wrote about how bodily dysfunction, and not appearance changes, had a negative impact on their identity. For example, loss of one’s own typical laughter may compromise one’s identity. This may have to do with losing “uniqueness and differentiation from relevant others” [42]. The other mentioned identity threat was being rejected as a blood donor. Belonging to a social group is important for identity [42]. The finding that identity in HNC can also be threatened by functional bodily changes extends other research that describes identity threat in HNC patients from an appearance perspective [14].

The present study revealed no relationship between body image and sexuality. This is somewhat surprising since a clear link between body image and poor sexual outcomes was found in other cancer populations [35]. Previous studies among HNC patients have reported conflicting results [43, 44]. A possible explanation for the lack of findings may be the use of only two (dichotomized) sexuality outcomes in the present study, for such a complex topic. This was necessary to be able to execute cross-gender analyses. Also, it could be that body image distress is more related to intimacy. This suggestion is supported by previous qualitative research [45]. HNC patients described how their changed body made them no longer feel sexually attractive and desired by their partner, which reduced the quality of the emotional connection. More research is warranted to unravel the relationship—if any—between body image and sexuality in HNC patients. For those studies, it is suggested to examine sexuality elaborately by using sexuality subscales and to incorporate instruments that measure intimacy.

The present study has some strengths and limitations. A strength is that we included a large sample of HNC patients, with a broad range of tumor sites and treatment modalities. However, due to the moderate response rate (45%), the results of the present study should be interpreted cautiously. Another limitation is that we used the dichotomized BIS as an outcome variable since no validated cut-off score is available. We dealt with this by using the most frequently used cut-off points (i.e., 8 and 10).

For clinical practice, it is recommended to identify HNC patients who suffer from body image distress, which can be monitored by letting patients complete PROMs when visiting the clinic. In that way, problems can be detected in a timely manner and supportive care provided as needed. Because evidence on effective supportive care targeting body image distress in HNC patients is still scarce [35], more research is needed.

## Conclusions

The prevalence of body image distress among HNC patients in the present study was 13–20%. Patients who are younger, those who had extensive surgery, problems with wound healing, symptoms of depression, or problems with social contact are more likely to have body image distress. HNC patients had most negative body image experiences in the area of social functioning.

## Electronic supplementary material

ESM 1 (DOCX 20 kb)

ESM 2 (DOCX 20 kb)

## References

[1] Verdonck-de Leeuw IM, Buffart LM, Heymans MW, Rietveld DH, Doornaert P, de Bree R, Buter J, Aaronson NK, Slotman BJ, Leemans CR, Langendijk JA (2014). The course of health-related quality of life in head and neck cancer patients treated with chemoradiation: a prospective cohort study. Radiother Oncol.

[2] Fingeret MC, Hutcheson KA, Jensen K, Yuan Y, Urbauer D, Lewin JS (2013). Associations among speech, eating, and body image concerns for surgical patients with head and neck cancer. Head Neck.

[3] Dropkin MJ (1989). Coping with disfigurement and dysfunction after head and neck cancer surgery: a conceptual framework. Semin Oncol Nurs.

[4] Hung TM, Lin CR, Chi YC, Lin CY, Chen EY, Kang CJ, Huang SF, Juang YY, Huang CY, Chang JT (2017). Body image in head and neck cancer patients treated with radiotherapy: the impact of surgical procedures. Health Qual Life Outcomes.

[5] Rhoten BA, Murphy B, Ridner SH (2013). Body image in patients with head and neck cancer: a review of the literature. Oral Oncol.

[6] Katz MR, Irish JC, Devins GM, Rodin GM, Gullane PJ (2000). Reliability and validity of an observer-rated disfigurement scale for head and neck cancer patients. Head Neck.

[7] White CA (2000). Body image dimensions and cancer: a heuristic cognitive behavioural model. Psychooncology.

[8] Katre C, Johnson IA, Humphris GM, Lowe D, Rogers SN (2008). Assessment of problems with appearance, following surgery for oral and oro-pharyngeal cancer using the University of Washington Appearance Domain and the Derriford Appearance Scale. Oral Oncol.

[9] Fingeret MC, Vidrine DJ, Reece GP, Gillenwater AM, Gritz ER (2010). Multidimensional analysis of body image concerns among newly diagnosed patients with oral cavity cancer. Head Neck.

[10] Fingeret MC, Yuan Y, Urbauer D, Weston J, Nipomnick S, Weber R (2012). The nature and extent of body image concerns among surgically treated patients with head and neck cancer. Psycho-Oncol.

[11] Howren MB, Christensen AJ, Karnell LH, Funk GF (2013). Psychological factors associated with head and neck cancer treatment and survivorship: evidence and opportunities for behavioral medicine. J Consult Clin Psychol.

[12] Rhoten BA, Deng J, Dietrich MS, Murphy B, Ridner SH (2014). Body image and depressive symptoms in patients with head and neck cancer: an important relationship. Support Care Cancer.

[13] Yaron G, Meershoek A, Widdershoven G, van den Brekel M, Slatman J (2017). Facing a disruptive face: embodiment in the everyday experiences of “disfigured” individuals. Hum Stud.

[14] Yaron G, Meershoek A, Widdershoven G, Slatman J (2018). Recognizing difference: in/visibility in the everyday life of individuals with facial limb absence. Disabil Soc.

[15] Verdonck-de Leeuw IM, Jansen F, Brakenhoff RH, Langendijk JA, Takes R, Terhaard CHJ, de Jong RJB, Smit JH, Leemans CR (2019). Advancing interdisciplinary research in head and neck cancer through a multicenter longitudinal prospective cohort study: the NETherlands QUality of life and BIomedical cohort (NET-QUBIC) data warehouse and biobank. BMC Cancer.

[16] Przezdziecki A, Alcorso J, Sherman KA (2016). My changed body: background, development and acceptability of a self-compassion based writing activity for female survivors of breast cancer. Patient Educ Couns.

[17] Hopwood P, Fletcher I, Lee A, Al Ghazal S (2001). A body image scale for use with cancer patients. Eur J Cancer.

[18] Melissant HC, Neijenhuijs KI, Jansen F, Aaronson NK, Groenvold M, Holzner B, Terwee CB, van Uden-Kraan CF, Cuijpers P, Verdonck-de Leeuw IM (2018). A systematic review of the measurement properties of the body image scale (BIS) in cancer patients. Support Care Cancer.

[19] van Verschuer VM, Vrijland WW, Mares-Engelberts I, Klem TM (2015). Reliability and validity of the Dutch-translated body image scale. Qual Life Res.

[20] Aaronson NK, Ahmedzai S, Bergman B, Bullinger M, Cull A, Duez NJ, Filiberti A, Flechtner H, Fleishman SB, Haes JCJM, Kaasa S, Klee M, Osoba D, Razavi D, Rofe PB, Schraub S, Sneeuw K, Sullivan M, Takeda F (1993). The European Organization for Research and Treatment of Cancer QLQ-C30: a quality-of-life instrument for use in international clinical trials in oncology. J Natl Cancer Inst.

[21] Singer S, Amdal CD, Hammerlid E, Tomaszewska IM, Castro Silva J, Mehanna H, Santos M, Inhestern J, Brannan C, Yarom N, Fullerton A, Pinto M, Arraras JI, Kiyota N, Bonomo P, Sherman AC, Baumann I, Galalae R, Fernandez Gonzalez L, Nicolatou-Galitis O, Abdel-Hafeez Z, Raber-Durlacher J, Schmalz C, Zotti P, Boehm A, Hofmeister D, Krejovic Trivic S, Loo S, Chie W-C, Bjordal K, Brokstad Herlofson B, Grégoire V, Licitra L, Life obotEQo, Head tE, Groups NC (2019). International validation of the revised European Organisation for Research and Treatment of Cancer Head and Neck Cancer module, the EORTC QLQ-HN43: phase IV. Head Neck.

[22] Isidori AM, Pozza C, Esposito K, Giugliano D, Morano S, Vignozzi L, Corona G, Lenzi A, Jannini EA (2010). Development and validation of a 6-item version of the female sexual function index (FSFI) as a diagnostic tool for female sexual dysfunction. J Sex Med.

[23] Rosen RC, Cappelleri JC, Smith MD, Lipsky J, Peña BM (1999). Development and evaluation of an abridged, 5-item version of the international index of erectile function (IIEF-5) as a diagnostic tool for erectile dysfunction. Int J Impot Res.

[24] Raes F, Pommier E, Neff KD, Van Gucht D (2011). Construction and factorial validation of a short form of the self-compassion scale. Clin Psychol Psychother.

[25] Zigmond A, Snaith R (1983). The hospital anxiety and depression scale. Acta Psychiatr Scand.

[26] Fayers P, Bottomley A (2002). Quality of life research within the EORTC—the EORTC QLQ-C30. Eur J Cancer.

[27] ter Kuile MM, Brauer M, Laan E (2006). The Female Sexual Function Index (FSFI) and the Female Sexual Distress Scale (FSDS): psychometric properties within a Dutch population. J Sex Marital Ther.

[28] Utomo E, Blok BF, Pastoor H, Bangma CH, Korfage IJ (2015). The measurement properties of the five-item International Index of Erectile Function (IIEF-5): a Dutch validation study. Andrology.

[29] Spinhoven P, Ormel J, Sloekers PP, Kempen GI, Speckens AE, Van Hemert AM (1997). A validation study of the hospital anxiety and depression scale (HADS) in different groups of Dutch subjects. Psychol Med.

[30] Falk Dahl CA, Reinertsen KV, Nesvold I-L, Fosså SD, Dahl AA (2010). A study of body image in long-term breast cancer survivors. Cancer.

[31] Giesinger JM, Kieffer JM, Fayers PM, Groenvold M, Petersen MA, Scott NW, Sprangers MA, Velikova G, Aaronson NK (2016). Replication and validation of higher order models demonstrated that a summary score for the EORTC QLQ-C30 is robust. J Clin Epidemiol.

[32] Henry M, Habib LA, Morrison M, Yang JW, Li XJ, Lin S, Zeitouni A, Payne R, MacDonald C, Mlynarek A, Kost K, Black M, Hier M (2014). Head and neck cancer patients want us to support them psychologically in the posttreatment period: survey results. Palliat Support Care.

[33] Braun V, Clarke V (2006). Using thematic analysis in psychology. Qual Res Psychol.

[34] Tong A, Sainsbury P, Craig J (2007). Consolidated criteria for reporting qualitative research (COREQ): a 32-item checklist for interviews and focus groups. Int J Qual Health Care.

[35] Fingeret MC, Teo I (2018). Body image care for cancer patients.

[36] Branch L, Feuz C, McQuestion M (2017). An investigation into body image concerns in the head and neck cancer population receiving radiation or chemoradiation using the body image scale: a pilot study. Journal of Medical Imaging and Radiation Sciences.

[37] Chen SC, Huang CY, Huang BS, Lin CY, Fan KH, Chang JT, Wu SC, Lai YH (2018). Factors associated with healthcare professional’s rating of disfigurement and self-perceived body image in female patients with head and neck cancer. European Journal of Cancer Care.

[38] Chen SC, Yu PJ, Hong MY, Chen MH, Chu PY, Chen YJ, Wang CP, Lai YH (2015). Communication dysfunction, body image, and symptom severity in postoperative head and neck cancer patients: factors associated with the amount of speaking after treatment. Support Care Cancer.

[39] Ellis MA, Sterba KR, Day TA, Marsh CH, Maurer S, Hill EG, Graboyes EM (2019). Body image disturbance in surgically treated head and neck cancer patients: a patient-centered approach. Otolaryng Head Neck.

[40] Hagedoorn M, Molleman E (2006). Facial disfigurement in patients with head and neck cancer: the role of social self-efficacy. Health Psychol.

[41] Pinto-Gouveia J, Duarte C, Matos M, Fraguas S (2014). The protective role of self-compassion in relation to psychopathology symptoms and quality of life in chronic and in cancer patients. Clin Psychol Psychother.

[42] Jaspal R (2012). Disfigurement: the challenges for identity and the strategies for coping. Psychol Stud.

[43] Gamba A, Romano M, Grosso LM, Tamburini M, Cantú G, Molinari R, Ventafridda V (1992). Psychosocial adjustment of patients surgically treated for head and neck cancer. Head Neck.

[44] Monga U, Tan G, Ostermann HJ, Monga TN (1997). Sexuality in head and neck cancer patients. Arch Phys Med Rehab.

[45] Rhoten BA, Sellers J, Charron E, Paul N, Radina ME (2019). Sexual activity after treatment for head and neck cancer: the experience of survivors. Cancer Nurs.

