# Supplementary material for: Body image distress in head and neck cancer patients: what are we looking at?
Source: Support Care Cancer. 2020 Sep 3;29(4):2161–9. doi: 10.1007/s00520-020-05725-1 (PMC7892513; doi:10.1007/s00520-020-05725-1)
Supplement: Supplementary file 1 — (DOCX 20 kb) [file 520_2020_5725_MOESM1_ESM.docx]

**Supplementary file 1. Results of the univariate logistic regression analyses.**

Manuscript title: Body image distress in head and neck cancer patients: what are we looking at?

Journal: Supportive Care in Cancer

Authors: H.C. Melissant, F. Jansen*, S.E. Eerenstein, P. Cuijpers, E. Laan, B.I. Lissenberg-Witte, A.S. Schuit, K.A. Sherman, C.R. Leemans, I.M. Verdonck-de Leeuw

*Corresponding author:

Femke Jansen

Amsterdam UMC, Vrije Universiteit Amsterdam, Department of Otolaryngology-Head and Neck Surgery, P.O. Box 7057, 1007 MB Amsterdam, Netherlands
Tel: +31 20 444 0681

E-mail: f.jansen1@amsterdamumc.nl

| **Variable** | **Mean (SD)** | **OR [95% CI]** | **Sig.** |
| --- | --- | --- | --- |
| Age |  | 0.96 [0.93-0.99] | 0.014 |
| Gender |  |  | 0.004 |
| Female |  | 1 |  |
| Male |  | 0.38 [0.20-0.73] |  |
| Married/ in a relationship |  |  | 0.060 |
| Yes |  | 1 |  |
| No |  | 1.94 [0.97-3.86] |  |
| Education level |  |  | 0.016 |
| Lower |  | 1 |  |
| Middle |  | 0.46 [0.21-0.99] |  |
| Higher |  | 0.27 [0.11-0.67] |  |
| Work situation |  |  | 0.845 |
| Employed |  | 1 |  |
| Unemployed/retired |  | 1.07 [0.53-2.20] |  |
| Tumor site |  |  | 0.47 |
| Oral cavity |  | 1 |  |
| Oropharynx |  | 1.21 [0.48-3.06] |  |
| Hypopharynx |  | 1.37 [0.31-5.99] |  |
| Larynx |  | 0.59 [0.21-1.61] |  |
| Other |  | 1.41 [0.54-3.65] |  |
| Tumor stage |  |  | 0.234 |
| I/II |  | 1 |  |
| III/IV |  | 1.50 [0.77-2.94] |  |
| Time since treatment |  | 0.94 [0.74-1.19] | 0.592 |
| Treatment modality |  |  | 0.008 |
| Surgery |  | 1 |  |
| Radiotherapy |  | 2.28 [0.70-7.48] |  |
| Chemoradiotherapy |  | 2.78 [0.88-8.75] |  |
| Surgery plus (chemo)radiotherapy |  | 5.58 [1.97-15.81] |  |
| Surgery extent ^a^ |  |  | 0.043 |
| Very large |  | 1 |  |
| Large |  | 0.38 [0.12-1.13] |  |
| Moderate |  | 0.23 [0.06-0.83] |  |
| Small |  | 0.23 [0.07-0.77] |  |
| Reconstruction |  |  | 0.524 |
| None |  | 1 |  |
| Primary closure |  | 1.71 [0.60-4.89] |  |
| Surgery with reconstruction |  | 1.75 [0.60-5.14] |  |
| Neck surgery |  |  | 0.086 |
| No |  | 1 |  |
| Yes |  | 2.11 [0.90-4.94] |  |
| HPV ^b^ |  |  | 0.934 |
| Negative |  | 1 |  |
| Positive |  | 1.07 [0.24-4.66] |  |
| EORTC QLQ-C30 summary score ^c^ | 84 (14) | 0.45 [0.34-0.59] | <0.001 |
| EORTC QLQ-HN43 ^c^ |  |  |  |
| Fear of progression | 23 (23) | 1.46 [1.26-1.69] | <0.001 |
| Dry mouth and sticky saliva | 33 (30) | 1.19 [1.07-1.32] | 0.001 |
| Pain in the mouth | 14 (19) | 1.32 [1.13-1.54] | <0.001 |
| Problems with senses | 19 (27) | 1.15 [1.03-1.28] | 0.012 |
| Problems with shoulder | 15 (26) | 1.22 [1.10-1.37] | <0.001 |
| Skin problems | 11 (18) | 1.22 [1.04-1.43] | 0.017 |
| Social eating | 16 (25) | 1.28 [1.14-1.44] | <0.001 |
| Speech | 22 (26) | 1.25 [1.12-1.40] | <0.001 |
| Swallowing | 15 (21) | 1.29 [1.12-1.48] | <0.001 |
| Problems with teeth | 16 (23) | 1.24 [1.08-1.41] | 0.002 |
| Coughing | 21 (28) | 1.20 [1.08-1.34] | 0.001 |
| Swelling in the neck | 10 (22) | 1.17 [1.03-1.33] | 0.015 |
| Neurological problems | 22 (31) | 1.16 [1.06-1.28] | 0.002 |
| Trismus | 19 (29) | 1.21 [1.10-1.34] | <0.001 |
| Problems with social contact | 4 (15) | 1.62 [1.30-2.03] | <0.001 |
| Weight loss | 10 (24) | 1.18 [1.06-1.33] | 0.004 |
| Problems with wound healing | 8 (20) | 1.34 [1.16-1.55] | <0.001 |
| Sexuality  FSFI-6  IIEF-5 | 13 (8)  12 (9) |  | 0.505 |
| No sexual activity | 43% | 1 |  |
| Sexually active without sexual problems | 26% | 0.80 [0.32-2.02] |  |
| Sexually active with sexual problems | 32% | 1.38 [0.62-3.04] |  |
| SCS-SF | 4.9 (0.9) | 0.38 [0.25-0.58] | <0.001 |
| HADS |  |  |  |
| HADS total | 9 (7) | 1.18 [1.12-1.24] | <0.001 |
| HADS depression | 4 (4) | 1.40 [1.26-1.55] | <0.001 |
| HADS anxiety | 5 (4) | 1.26 [1.16-1.37] | <0.001 |

*EORTC QLQ-C30/HN43* 30-item core European Organisation for Research and Treatment of Cancer Quality of Life Questionnaire/head and neck cancer, 43 items, *HADS* Hospital Anxiety and Depression Scale, *SCS-SF* Self Compassion Scale – Short Form. ^a^ Small: C02-laser of vocal fold, lip excision, ear amputation, skin excision small nose tumor. Moderate: excision of sublingual/submandibular salivary gland, transoral excision, lip surgery with reconstruction, partial sinus resection, skin excision with local reconstruction, neck surgery. Large: parotidectomy with neck surgery, marginal and segmental mandibular resection, transoral excision with reconstruction, extensive sinus surgery, maxillectomy, skin excision with neck surgery or reconstruction. Very large: commando procedure, laryngectomy, lateral temporal bone surgery. ^b^ n=54 oropharyngeal cancer patients with a known HPV status. ^c^ OR per 10 point increase in subscale.
