# Supplementary material for: Body image distress in head and neck cancer patients: what are we looking at?
Source: Support Care Cancer. 2020 Sep 3;29(4):2161–9. doi: 10.1007/s00520-020-05725-1 (PMC7892513; doi:10.1007/s00520-020-05725-1)
Supplement: Supplementary file 2 — (DOCX 20 kb) [file 520_2020_5725_MOESM2_ESM.docx]

**Supplementary file. COREQ (COnsolidated criteria for REporting Qualitative research) Checklist**

Manuscript title: Body image distress in head and neck cancer patients: what are we looking at?

Journal: Supportive Care in Cancer

Authors: H.C. Melissant, F. Jansen*, S.E. Eerenstein, P. Cuijpers, E. Laan, B.I. Lissenberg-Witte, A.S. Schuit, K.A. Sherman, C.R. Leemans, I.M. Verdonck-de Leeuw

*Corresponding author:

Femke Jansen

Amsterdam UMC, Vrije Universiteit Amsterdam, Department of Otolaryngology-Head and Neck Surgery, P.O. Box 7057, 1007 MB Amsterdam, Netherlands

Tel: +31 20 444 0681

E-mail: f.jansen1@amsterdamumc.nl

| **Topic** | **Item No.** | **Guide Questions/Description** | **Reported on Page No.** |
| --- | --- | --- | --- |
| **Domain 1: Research team and reflexivity** | | | |
| *Personal characteristics* | | | |
| Interviewer/facilitator | 1 | Which author/s conducted the interview or focus group? | Heleen C Melissant  Anouk S Schuit |
| Credentials | 2 | What were the researcher’s credentials? E.g. PhD, MD | Heleen C Melissant, MSc. PhD candidate  Femke Jansen, PhD. Senior researcher  Simone E.J. Eerenstein, PhD. MD.  Pim Cuijpers, PhD. Professor.  Ellen Laan, PhD. Professor.  Birgit I Lissenberg-Witte, PhD. Senior researcher.  Anouk S Schuit, MSc. PhD candidate.  Kerry A. Sherman, PhD. Professor.  C. René Leemans, PhD., MD. Professor.  Irma M Verdonck-de Leeuw, PhD. Professor. |
| Occupation | 3 | What was their occupation at the time of the study? | PhD candidate, Senior researcher, Professor. |
| Gender | 4 | Was the researcher male or female? | First author: female |
| Experience and training | 5 | What experience or training did the researcher have? | The first author participated in a qualitative research interview training in the Netherlands in 2016. She conducted interviews and analyzed qualitative data in 3 other studies that were published in international peer-reviewed scientific journals. |
| *Relationship with participants* |  |  |  |
| Relationship established | 6 | Was a relationship established prior to study commencement? | No |
| Participant knowledge of the interviewer | 7 | What did the participants know about the researcher? e.g. personal goals, reasons for doing the research | Information about the research goal was included in the participant information letter and informed consent form. Participants were aware that the study was part of a PhD project. |
| Interviewer characteristics | 8 | What characteristics were reported about the interviewer/facilitator? e.g. Bias, assumptions, reasons and interests in the research topic | N/A |
| **Domain 2: Study design** | | | |
| *Theoretical framework* | | | |
| Methodological orientation and Theory | 9 | What methodological orientation was stated to underpin the study? e.g. grounded theory, discourse analysis, ethnography, phenomenology, content analysis | See methods section |
| *Participant selection* | | | |
| Sampling | 10 | How were participants selected? e.g. purposive, convenience, consecutive, snowball | See methods section |
| Method of approach | 11 | How were participants approached? e.g. face-to-face, telephone, mail, email | See methods section |
| Sample size | 12 | How many participants were in the study? | See results section |
| Non-participation | 13 | How many people refused to participate or dropped out? Reasons? | See results section |
| *Setting* | | | |
| Setting of data collection | 14 | Where was the data collected? e.g. home, clinic, workplace | See methods section |
| Presence of non-participants | 15 | Was anyone else present besides the participants and researchers? | N/A |
| Description of sample | 16 | What are the important characteristics of the sample? e.g. demographic data, date | See table 1. Patient characteristics |
| *Data collection* | | | |
| Interview guide | 17 | Were questions, prompts, guides provided by the authors? Was it pilot tested? | N/A |
| Repeat interviews | 18 | Were repeat interviews carried out? If yes, how many? | N/A |
| Audio/visual recording | 19 | Did the research use audio or visual recording to collect the data? | N/A |
| Field notes | 20 | Were field notes made during and/or after the interview or focus group? | N/A |
| Duration | 21 | What was the duration of the interviews or focus group? | N/A  79% of participants finished the total writing intervention between 15-60 minutes. |
| Data saturation | 22 | Was data saturation discussed? | N/A |
| Transcripts returned | 23 | Were transcripts returned to participants for comment and/or correction? | N/A |
| **Domain 3: Analysis and findings** | | | |
| *Data analysis* | | | |
| Number of data coders | 24 | How many data coders coded the data? | 2 coders |
| Description of the coding tree | 25 | Did authors provide a description of the coding tree? | See table 3: Qualitative results. |
| Derivation of themes | 26 | Were themes identified in advance or derived from the data? | See methods section: qualitative analysis |
| Software | 27 | What software, if applicable, was used to manage the data? | Atlas.ti 8 |
| Participant checking | 28 | Did participants provide feedback on the findings? | No |
| *Reporting* | | | |
| Quotations presented | 29 | Were participant quotations presented to illustrate the themes/findings? Was each quotation identified? e.g. participant number | Yes - Quotations were presented. Quotations were not identified with a participant number. |
| Data and findings consistent | 30 | Was there consistency between the data presented and the findings? | Yes – See results section/Table 3 |
| Clarity of major themes | 31 | Were major themes clearly presented in the findings? | Yes – See results section/Table 3 |
| Clarity of minor themes | 32 | Is there a description of diverse cases or discussion of minor themes? | Yes – See results section/Table 3 |

Developed from: Tong A, Sainsbury P, Craig J. Consolidated criteria for reporting qualitative research (COREQ): a 32-item checklist for interviews and focus groups. *International Journal for Quality in Health Care*. 2007. Volume 19, Number 6: pp. 349 – 357.
